# Staphylococcal early-onset prosthetic valve endocarditis: a condition bound for surgery

**DOI:** 10.1186/s12879-025-11999-9

**Published:** 2025-12-19

**Authors:** Antonio de Santis, Eduardo Cukierkorn, Flavio Tarasoutchi, Roney Orismar Sampaio, Tshimbalanga Merite, Milena R. Paixão, Carlos Manuel A. Brandão, Elinthon T. Veronese, Francisco Monteiro de Almeida Magalhães, Tarso A. D. Accorsi, Guilherme S. Spina, Tania Mara Varejão Strabelli, Rinaldo Focaccia Siciliano

**Affiliations:** 1 Heart Valve Disease Division, Heart Institute (InCor), University of São Paulo Medical School, Av. Dr. Eneas de Carvalho Aguiar, Sp São Paulo, 05403-000 Brasil; 2 Cardiology Department, Heart Institute (InCor), University of São Paulo Medical School, São Paulo, Brazil; 3 Cardiac Surgery Department, Heart Institute (InCor), University of São Paulo Medical, São Paulo, Brazil; 4 Infection Control Team, Heart Institute (InCor), University of São Paulo Medical, São Paulo, Brazil

**Keywords:** Endocarditis, Heart valve disease valve, Valve heart surgery

## Abstract

**Background:**

Early-onset prosthetic valve endocarditis (EO-PVE) is linked to poor in-hospital outcomes. Staphylococcus spp. poses a significant concern due to its higher mortality rates compared to other major infectious agents.

**Objectives:**

Provide a more detailed, comprehensive evaluation of the clinical characteristics and in-hospital mortality predictors related to staphylococcal EO-PVE.

**Methods:**

This observational, retrospective, single-center study was conducted at a tertiary hospital in Brazil from 1997 to 2019, spanning a 22-year period. A total of 105 consecutive cases of left-heart staphylococcal EO-PVE were analyzed.

**Results:**

There was a predominance of coagulase-negative staphylococci prosthetic valve endocarditis (CoNS PVE) over Staphylococcus aureus prosthetic valve endocarditis (SAPVE) (76% and 24%, respectively). Prosthetic valve replacement for EO-PVE treatment was performed in 73% of cases. In-hospital mortality was 49%, with SAPVE associated with a higher in-hospital mortality than CoNS PVE (80% versus 43%, *p* < 0.001). In-hospital mortality predictors identified by univariate analysis included older age (*p* < 0.001), aortic prosthetic endocarditis (*p* < 0.001), peri-annular abscess (*p* = 0.002), SAPVE (*p* < 0.001), NYHA functional class III/IV (*p* = 0.02), previous combined myocardial revascularization with valve replacement surgery (*p* = 0.02), left ventricular dysfunction (*p* < 0.001), leukocytosis (*p* = 0.02), and higher C-reactive protein levels (*p* = 0.006). In a multivariate analysis, SAPVE was identified as an independent risk factor for in-hospital mortality (odds ratio [OR] 10.2; *p* = 0.006), whereas prosthetic valve replacement was associated with improved in-hospital survival (OR 0.2; *p* = 0.04).

**Conclusion:**

Staphylococcal EO-PVE is associated with increased in-hospital mortality, particularly in SAPVE cases. In this study, all non-operated SAPVE patients died primarily due to fulminant septic shock. Prosthetic valve replacement was significantly linked to in-hospital survival, and only 5.7% of the study population survived without cardiac surgical intervention.

**Clinical trial number:**

Not applicable.

**Supplementary Information:**

The online version contains supplementary material available at 10.1186/s12879-025-11999-9.

## Introduction

Prosthetic valve endocarditis (PVE) occurs in 3–6% of patients within five years of valve replacement surgery and carries significant morbidity and in-hospital mortality [1, 2]. Early-onset prosthetic valve endocarditis (EO-PVE) is defined as infection diagnosed within one year after heart valve surgery [3]. The clinical use of this classification is justified by the distinct microbiological profile of this period, characterized by a higher prevalence of virulent hospital-acquired pathogens. Among these, *Staphylococcus aureus* is particularly notable for its aggressiveness, with mortality rates of up to 48.5% and frequent embolic complications affecting the central nervous system (CNS), spleen, kidneys, and peripheral arteries [4]. Previous studies demonstrate that *S. aureus* prosthetic infective endocarditis (SAPVE) is an independent predictor of in-hospital mortality [5]

Due to the complexity of decision-making in EO-PVE—often influenced by the severity of the clinical state and complications such as CNS embolism—management must be undertaken by a dedicated endocarditis team. Regarding therapy, most recommendations derive from relatively small retrospective studies, which consistently suggest higher mortality rates in non-operated patients. This study, therefore, aims to describe the clinical characteristics, in-hospital mortality predictors, and the role of surgical treatment in staphylococcal EO-PVE. The study population comprises exclusively left-sided EO-PVE, given its clinical significance and frequency, since tricuspid and pulmonary prostheses are uncommon.

## Methods

**Fig. 1:**
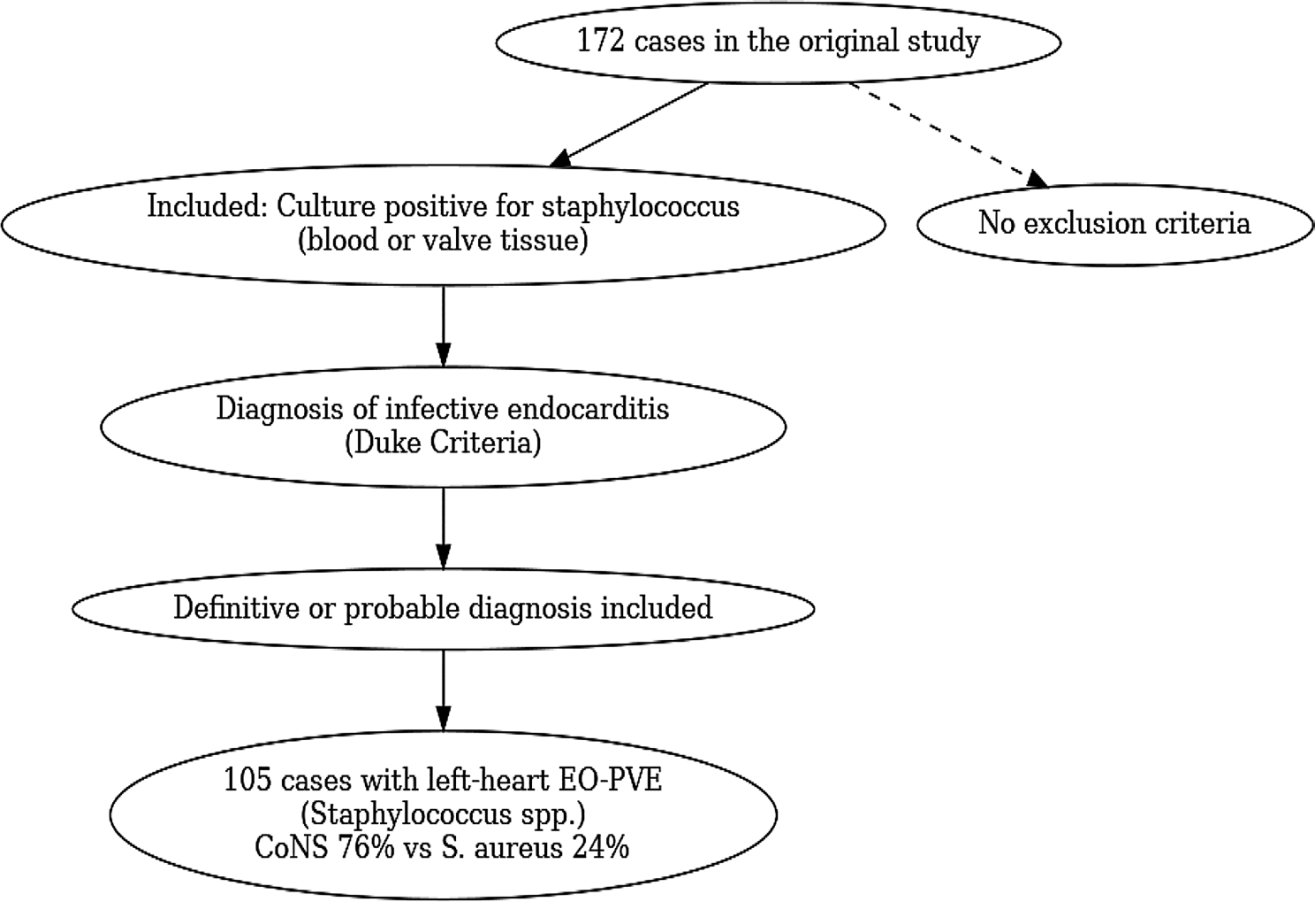
Flowchart of inclusion criteria. Of the 172 cases from the original study, 105 fulfilled criteria for staphylococcal-related early-onset prosthetic valve endocarditis (EO-PVE), with distribution according to *staphylococcus aureus* and coagulase-negative staphylococci (CoNS)

The current study is a sub-analysis of a historical cohort published by Siciliano et al. [3], which evaluated prosthetic valve endocarditis (PVE) at the Heart Institute (InCor), University of São Paulo Medical School, Brazil. The original study included patients diagnosed between 1997 and 2014, representing more than a decade of experience with PVE during the first postoperative year. Its primary objective was to assess the annual incidence of PVE within the first year after valve surgery and its microbiological profile. Diagnosis was established according to the Modified Duke Criteria (2000), with microorganisms identified through blood cultures and cultures of excised valve tissue. In contrast to the original study, the present analysis focused exclusively on staphylococcal early-onset PVE (EO-PVE), given its virulence and distinct clinical importance (Fig. 1). Only patients with culture-positive staphylococcal endocarditis were included

For the present analysis, only patients with culture-positive staphylococcal endocarditis were included, given the virulence and distinct clinical importance of this group of microorganisms (Fig. 1). A total of 105 patients met the selection criteria: 103 fulfilled the definition of definite endocarditis, and two were classified as possible endocarditis. Among the included cases, coagulase-negative staphylococci (CoNS) were predominant (76%), while *Staphylococcus aureus* accounted for 24%. According to the Modified Duke Criteria, definite endocarditis requires either two major criteria, one major plus three minor criteria, or five minor criteria. Possible endocarditis was defined by the presence of one major plus one minor criterion or three minor criteria. For this analysis, both definite and probable cases were included. To ensure a homogeneous population, only left-sided prosthetic valve endocarditis was analyzed, given its higher prevalence and predominance in the cohort. The primary objective of this sub-analysis was to identify variables associated with poor prognosis in patients with staphylococcal PVE.

All surgical indications were determined by a dedicated multidisciplinary endocarditis team, composed of clinical cardiologists, cardiac surgeons, infectious disease specialists, intensive care physicians, and emergency physicians.

The primary outcome was in-hospital mortality. Demographic information, type of valve prosthesis (mechanical or biological), laboratory findings, microbiological profile, echocardiographic characteristics, clinical features, and data on prosthetic valve replacement surgery were also collected. For the multivariate model, only variables with statistical significance in the univariate analysis were included.

The available clinical and surgical information was limited to the original study database, which did not include details on previous sternotomies, operative time, or perioperative complications. Nevertheless, it was possible to calculate the pre-surgical Risk-E score (Risk Score for Cardiac Surgery in Active Infective Endocarditis) in 34 patients, providing valuable prognostic information despite these constraints. The results are presented in Table 1.

**Table 1:** Demographic data

	All patients (*n* = 105)
Mean age (years)	51.3 ± 16.4
Male sex	67 (63%)
In-hospital mortality	52 (49%)
Exclusive aortic endocarditis	61 (58%)
Exclusive mitral endocarditis	39 (37.2%)
Peri-annular abscess	34 (32%)
Aorto-cavitary fistula	3 (2.8%)
MSSA	10 (9.5%)
MRSA	15 (14.2%)
CoNS	80 (76.1%)
Severe Heart Failure related to prosthetic valve dysfunction (NYHA III/IV)	22 (21%)
Systemic embolism	19 (18%)
Median time from valve replacement surgery to diagnosis of PVE (in days)	44 (25–93)
Vegetation	70 (66.6%)
Biologic prosthetic valve	98 (93.3%)
Combined mitral and aortic valve endocarditis	5 (4.8%)
Prosthetic valve replacement	77 (73%)
Definitive Duke criteria	103 (98%)
Median time from PVE diagnosis to prosthetic valve replacement (in days)	6 (3–12)

Data are expressed as mean ± standard deviation, median, and interquartile range or number (%). MSSA: methicillin-sensitive *Staphylococcus aureus*; MRSA: methicillin-resistant *Staphylococcus aureus*; CoNS PVE: Coagulase-Negative Staphylococci prosthetic valve endocarditis; NYHA: New York Heart Association; PVE: Prosthetic valve endocarditis

The primary indications for prosthetic valve replacement were severe heart failure related to prosthetic dysfunction, peri-annular abscess or aorto-cavitary fistulas, large mobile vegetations with systemic embolization ( > 10 mm), and persistent sepsis despite properly guided antibiotic therapy for more than 5–7 days [2, 6, 7]. Owing to the historical design of the study, surgical indications were based on the contemporary guidelines available at the time. A comparative discussion with the most recent ESC 2023 guidelines—classifying indications into heart failure, uncontrolled infection, and high risk of embolism or established embolism—has been added to the Discussion section. The local ethics committee approved the present study.

## Statistical analysis

All statistical analyses were performed with SPSS 21.0 statistical package (SPSS, Chicago, IL, USA). The distribution of numerical variables was assessed using the Kolmogorov–Smirnov test. Variables with a normal distribution are expressed as mean ± standard deviation, whereas variables with a non-normal distribution are presented as median and interquartile range (IQR). Qualitative variables are reported as numbers and percentages. In univariate analysis, comparisons between groups were made using the Student’s t-test or ANOVA for normally distributed variables, and the Mann–Whitney U or Kruskal–Wallis test for non-normally distributed variables, as appropriate. Categorical variables were compared using the chi-square test. For multivariate analysis, logistic regression analysis was used to predict the risk of in-hospital mortality. Values of *p* < 0.05 were considered statistically significant.

## Results

Among these patients, we found 105 cases with left-heart EO-PVE due to *Staphylococcus spp.,* with a predominance of Coagulase-Negative Staphylococci (CoNS) over *Staphylococcus aureus* (76% versus 24%, respectively).

**Fig. 2:**
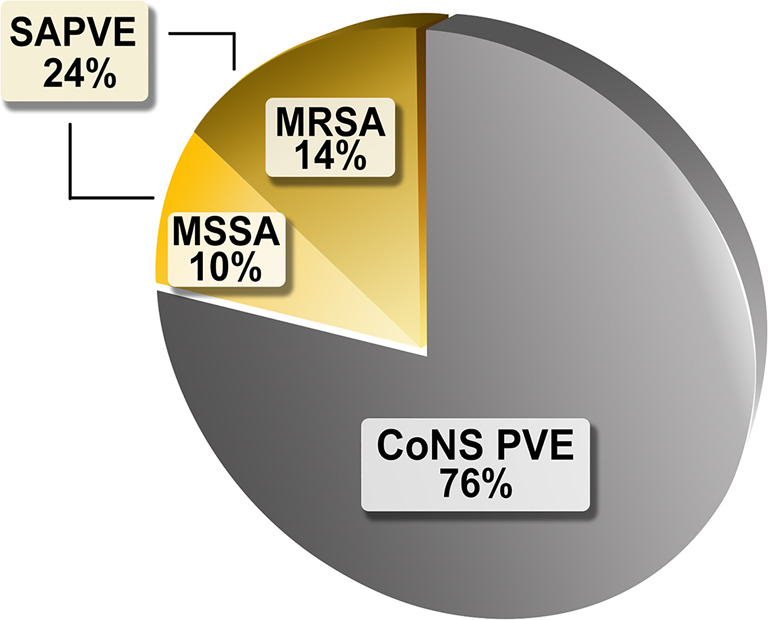
Etiologic distribution of staphylococcal early-onset prosthetic valve endocarditis. CoNS PVE: coagulase-negative staphylococci prosthetic valve endocarditis; MSSA: methicillin-sensitive *staphylococcus aureus*; MRSA: methicillin-resistant *staphylococcus aureus*; SAPVE: *staphylococcus aureus* prosthetic valve endocarditis

The final population consisted of 105 patients (mean age 51.3 ± 16.4 years, with 63% male sex) with left-heart Staphylococcal endocarditis. The median time from the previous valve replacement surgery to the PVE diagnosis was 44 days (25–93). There was a predominance of CoNS PVE (*n* = 80, 76%) over SAPVE (*n* = 25, 24%; 60% methicillin-resistant *Staphylococcus aureus [MRSA]*, 40% methicillin-sensitive *Staphylococcus aureus [MSSA]*), as represented in Fig. 2. With the present data, the team was unable to determine the different types of CONS.

Regarding the Duke criteria, 98% of the study population had definitive criteria. Prosthetic valve replacement for PVE treatment was performed in 73% of the patients, and the overall in-hospital mortality was 49%. The main complications were peri-annular abscess (32%); severe heart failure - New York Heart Association (NYHA) functional classification III/IV- related to prosthetic dysfunction (21%); systemic embolization (18%), and aorto-cavitary fistulas (3%). (Table 2). In comparison with CoNS PVE, SAPVE was associated with a higher in-hospital mortality (80% *versus* 43%, *p* < 0.001) and a trend towards earlier clinical presentation and diagnosis (median time from valve replacement to the PVE diagnosis: 33 days [18–94] *versus* 51.5 days [25.7–91.5], *p* = 0.05). However, there was a higher rate of prosthetic valve replacement for endocarditis treatment in CoNS PVE compared to SAPVE (83% versus 40%, *p* < 0.001, respectively) (Table 3). Only 5.7% (6/105) of patients were discharged from the hospital without surgical treatment.

**Table 2:** Comparison of demographic data between SAPVE and CoNS PVE

	All patients (*n* = 105)	Risk E Group (*n* = 34)	p-value
Mean age (years)	51.3 ± 16.4	57.2 ± 15.3	0.064
Male sex	67 (63.8%)	21 (61.8%)	0.992
In-hospital mortality	52 (49.5%)	16 (47.1%)	0.958
Exclusive aortic endocarditis	66 (62.9%)	24 (70.6%)	0.539
Exclusive mitral endocarditis	44 (41.9%)	12 (35.3%)	0.630
Peri-annular abscess	6 (5.7%)	3 (8.8%)	0.811
Aorto-cavitary fistula	34 (32.4%)	15 (44.1%)	0.299
MSSA	3 (2.9%)	1 (2.9%)	1.000
MRSA	70 (66.7%)	25 (73.5%)	0.592
CoNS	19 (18.1%)	6 (17.6%)	1.000
Severe Heart Failure related to prosthetic valve dysfunction (NYHA III/IV)	10 (9.5%)	4 (11.8%)	0.960
Systemic embolism	15 (14.3%)	2 (5.9%)	0.318
Median time from valve replacement surgery to diagnosis of PVE (in days)	80 (76.2%)	28 (82.4%)	0.608
Vegetation	25 (23.8%)	6 (17.6%)	0.608

Comparison between the overall cohort (*n* = 105) and the Risk-E subgroup (*n* = 34 patients with available perioperative data). No significant differences were observed between groups, supporting the representativeness of the Risk-E subgroup. Data are expressed as mean ± standard deviation, median (interquartile range), or number (%). Comparisons were performed using Student’s t test or Mann–Whitney U test for continuous variables, and chi-square or Fisher’s exact test for categorical variables, as appropriate

**Table 3:** Comparison of demographic data between SAPVE and CoNS PVE

	All patients (*n* = 105)	SAPVE (*n* = 25)	CoNS PVE (*n* = 80)	p-value
Mean age (years)	51.3 ± 16.4	55.3 ± 16.5	50 ± 16.3	0.16
Male sex	67 (63%)	16	51	0.98
In-hospital mortality	52 (49%)	20 (80%)	32 (43%)	0.001
Exclusive aortic endocarditis	61 (58%)	14	47	0.73
Exclusive mitral endocarditis	39 (37%)	10	29	0.80
Peri-annular abscess	34 (32%)	10	24	0.35
Severe heart failure related to prosthetic valve dysfunction (NYHA III/IV)	22 (21%)	6	16	0.5
Systemic embolism	19 (18%)	3	16	0.36
Median time from valve replacement surgery to diagnosis of PVE (in days)	44 (25–93)	33 (18–94)	51.5 (25.7–91.5)	0.05
Vegetation	70 (66.6%)	17	53	0.87
Biologic prosthetic valve	98 (93.3%)	23	75	0.75
Prosthetic valve replacement	77 (73%)	10 (40%)	67 (83%)	0.001
Definitive Duke criteria	103 (98%)	25 (100%)	78 (97%)	0.42
Median time from PVE diagnosis to prosthetic valve replacement (in days)	6 (3–12)	9 (5.2 -12,5)	6 (3–11)	0.35

Data are expressed as mean ± standard deviation, median, and interquartile range or number (%). SAPVE: *Staphylococcus aureus* prosthetic valve endocarditis; CoNS PVE: Coagulase-Negative Staphylococci prosthetic valve endocarditis; NYHA: New York Heart Association; PVE: Prosthetic valve endocarditis

**Fig. 3:**
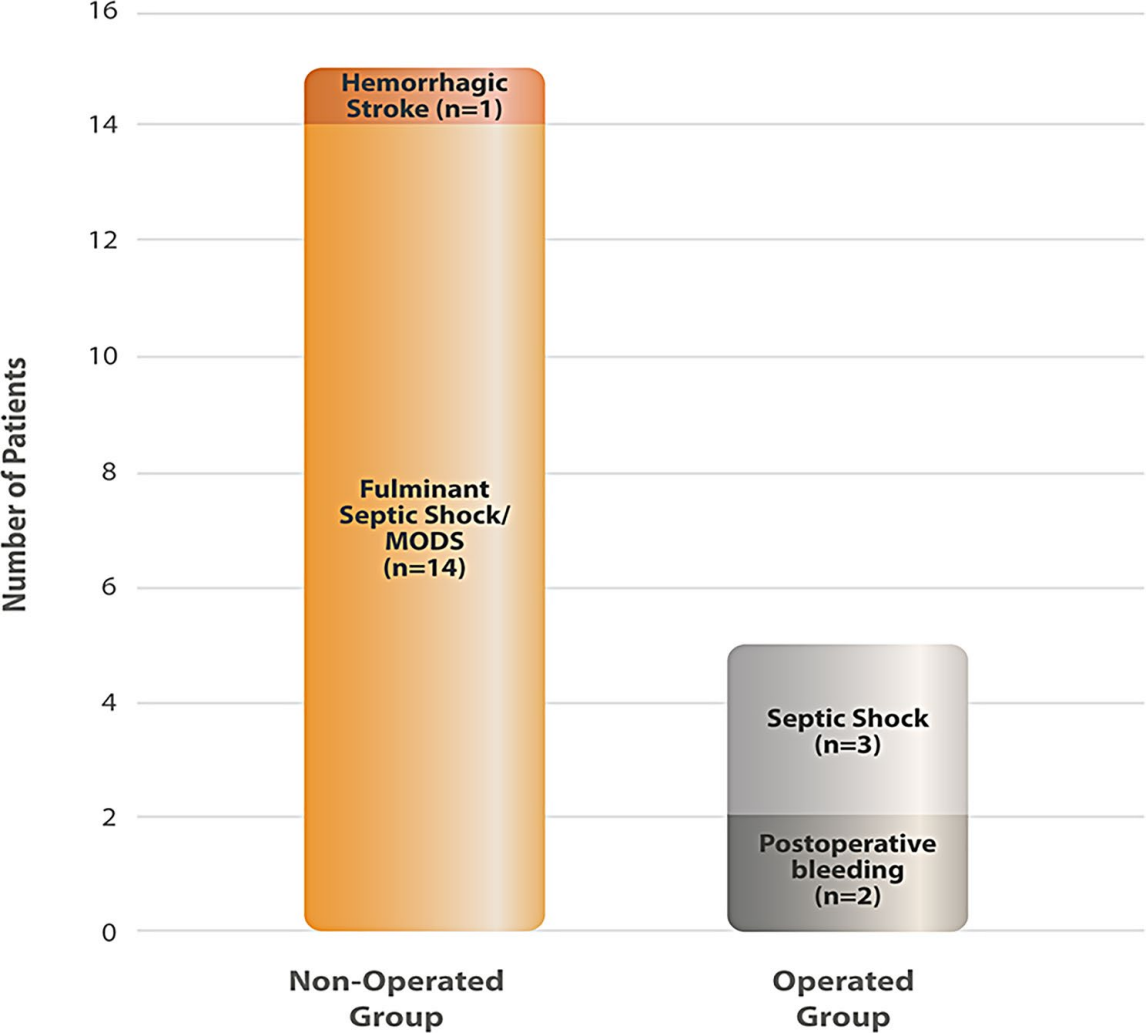
Histogram of death causes related to *staphylococcus aureus* prosthetic valve endocarditis (*n* = 20). In the non-operated group (*n* = 15), the major cause of death was fulminant septic shock with multiple organ dysfunction syndrome (MODS). In the operated group (*n* = 5), deaths were primarily related to postoperative septic shock and bleeding

In the SAPVE group, all patients who did not undergo prosthetic valve replacement died (*n* = 15). Despite surgical indications, 14 of these patients (93.3%) developed fulminant septic shock within 24 hours of hospital admission, leaving insufficient time for surgical planning, as illustrated in Fig. 3. Among those who underwent surgery, five patients died due to postoperative complications.

On univariate analysis, significant risk factors for in-hospital mortality included older age (*p* < 0.001), aortic prosthetic endocarditis (*p* < 0.001), periannular abscess (*p* = 0.002), SAPVE (*p* < 0.001), NYHA functional class III/IV (*p* = 0.02), previous combined coronary artery bypass-grafting (CABG) with valve replacement surgery (*p* = 0.02), left ventricular dysfunction (*p* < 0.001), leukocytosis (*p* = 0.02) and higher C-reactive protein levels (*p* = 0.006), as presented in Table 4. Additionally, prosthetic valve replacement was significantly associated with improved in-hospital survival (*p* < 0.001). The median time from PVE diagnosis to prosthetic valve replacement in the surgical group (*n* = 77) was 6 days (3–12), with no statistically significant difference between the deceased and alive groups.

**Table 4:** Univariate analysis of in-hospital mortality in staphylococcal PVE

	Alive (*n* = 53)	Dead (*n* = 52)	p-value
Age > 60 (years)	8 (15%)	25 (48%)	< 0.001
Male sex	33 (62.2%)	34 (65.3%)	0.73
Exclusive aortic endocarditis	25(47.1%)	41 (78.8%)	< 0.001
Previous combined valve replacement surgery with myocardial revascularization	3 (5.6%)	11 (21.1%)	0.02
Peri-annular abscess	10 (18.8%)	24 (46.1%)	0.002
SAPVE	5 (9.4%)	20 (38.4%)	< 0.001
Severe heart failure related to prosthetic valve dysfunction (NYHA III/IV)	19 (35.8%)	26 (50%)	0.02
Systemic embolism	7 (13.2%)	12 (23%)	0.18
Median time from valve replacement surgery to diagnosis of PVE (in days)	35 (22–83)	48 (29.7–99.2)	0.27
Vegetation	34 (64.1%)	36 (69.2%)	0.58
Biologic prosthetic valve	49 (92.4%)	49 (94.2%)	0.71
Prosthetic valve replacement for endocarditis treatment	47 (88.6%)	30 (57.6%)	< 0.001
Median time from PVE diagnosis to prosthetic valve replacement (in days)	7 (5–14.5)	5 (3–9)	0.06
LVEF (%)	61.9 ± 11.4	50.6 ± 18.4	< 0.001
Hemoglobin (mg/dL)	10.7 ± 1.96	10.3 ± 1.85	0.33
Leukocytes count (x10^3^ cells/mm^3^)	13 ± 6.17	16.2 ± 6.9	0.02
Platelet count (x10^3^ cells/mm3)	189.4 ± 81.2	153.9 ± 92.8	0.06
C-reactive protein (mg/L)	96.2 ± 45.7	183.5 ± 89.4	0.006

Data are expressed as mean ± standard deviation, median, and interquartile range or number (%). Laboratory data presented in the table represent preoperative values. LVEF: Left ventricular ejection fraction, measured preoperatively.; SAPVE: *Staphylococcus aureus* prosthetic valve endocarditis; NYHA: New York Heart Association; PVE: Prosthetic valve endocarditis

Multivariate analysis showed that SAPVE was an independent risk factor for in-hospital mortality (odds ratio [OR] 10.2; *p* = 0.006; 95% CI [1.89–55.2]) and reinforced the potential protective effect of prosthetic valve replacement in staphylococcal EO-PVE (OR 0.2; *p* = 0.04; 95% CI [0.04–0.93]).

## Discussion

Although rare, EO-PVE has a high potential for clinical complications and significant in-hospital mortality. From an etiopathogenic perspective, previous studies have demonstrated a predominance of staphylococcal infections in these scenarios, with a prevalence of nearly two-thirds of the cases [2, 8]. The present work was designed due to the clinical relevance and scarcity of previous studies focusing on this etiological subgroup of PVE. [4, 9, 10–13] While *Streptococcus spp.* and *Enterococcus spp.* are recognized as the second and third most frequent pathogens in infective endocarditis, the present study specifically aimed to evaluate staphylococcal EO-PVE, primarily because of its particularly aggressive clinical course.

Staphylococcal EO-PVE demonstrates a high virulence with an in-hospital mortality of 49% and high rates of clinical complications (peri-annular abscess-32%, heart failure-NYHA class III/IV-21%, systemic embolization-18%). Moreover, staphylococcal EO-PVE often requires a combination treatment strategy, including the association of proper antimicrobial therapy and valve replacement surgery (with a surgical rate of 73%).

**Fig. 4:**
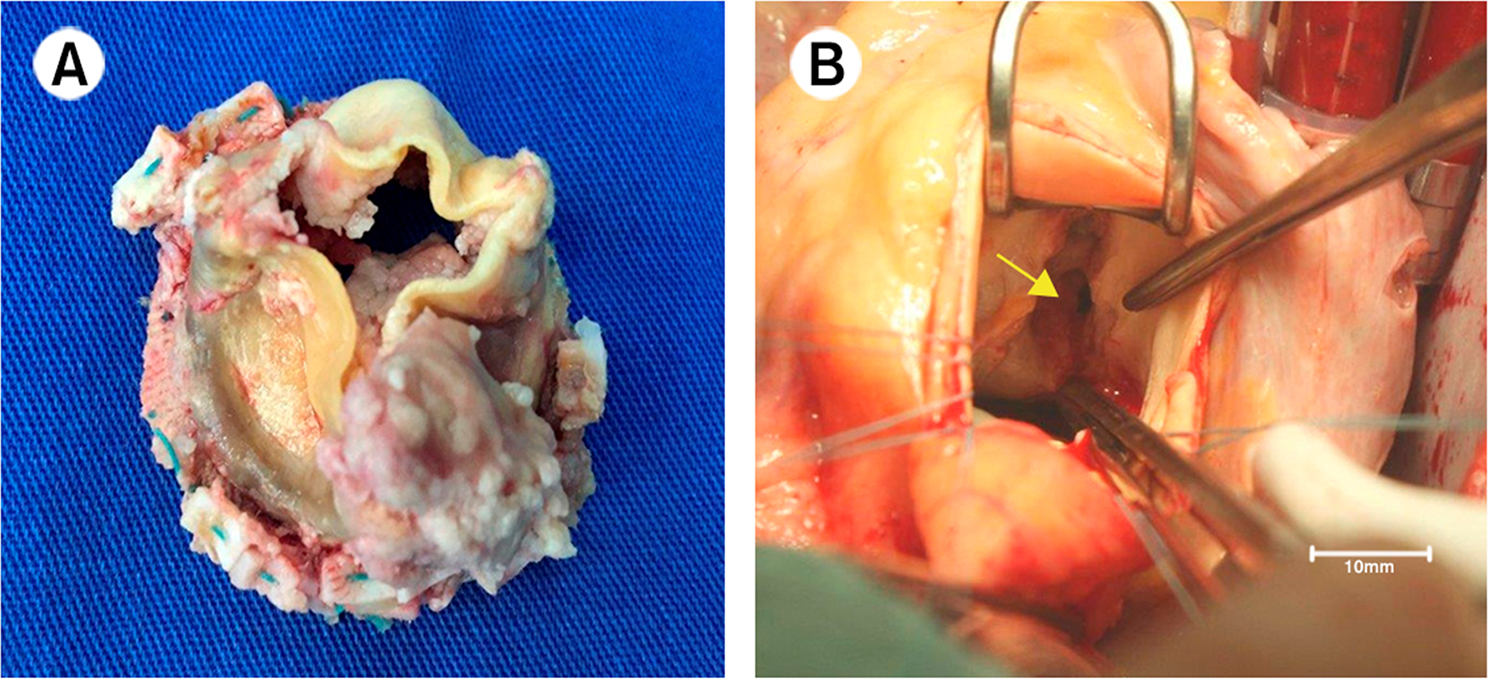
Staphylococcal early-onset prosthetic valve endocarditis examples. Panel **A**: multiple prosthetic vegetations with associated valvular dysfunction. Panel **B**: aorto-right ventricular fistula (yellow arrow)

Staphylococcal EO-PVE was diagnosed correctly, with 98% of the study population presenting definite endocarditis according to the Duke modified criteria. Vegetations were the most common echocardiographic feature (found in 66.6% of the cases). Fig. 4 represents multiple prosthetic vegetations in a staphylococcal EO-PVE (panel A) and an aorto-right ventricular fistula (panel B).

Regarding the staphylococcal species, coagulase-negative strains were predominant (76%) compared to S. aureus (24%), with some notable clinical differences between them. SAPVE presented a higher fatality rate than CoNS PVE (80% *versus* 43%, *p* < 0.001) and a propensity to earlier clinical presentation (median time from previous valve replacement surgery to the PVE diagnosis of 33 days [18–94]). Conversely, SAPVE had a lower rate of prosthetic valve replacement than CoNS PVE (40% *versus* 83%, *p* < 0.001). Despite the inherent limitations of an observational study, the high mortality associated with SAPVE may be related to these observed lower rates of surgical treatment. The specific analysis of deaths in the SAPVE group demonstrated that from the 15 non-operated cases, 14 (93.3%) were due to fulminant septic shock leading to multiple organ dysfunction syndrome (MODS) in less than 24 hours. Thus, despite clinical indication, there was no proper time to perform the prosthetic valve replacement. In current guidelines, one of the most frequent indications for valve replacement in infective endocarditis is a clinical condition known as uncontrolled infection, defined by persistent sepsis despite proper antibiotic therapy for more than seven days [2, 6, 7]. Reinforcing the aggressiveness of SAPVE with early-onset sepsis, our study suggests that the time window for proper surgical intervention in these situations could be narrower than in other etiologies. The definition of the standard 7-day period for treatment failure appears to be overly compliant when considering such an inexorable progression. Early prosthetic valve replacement, before clinical irreversibility, may be crucial in obtaining favorable outcomes. In this sepsis scenario, antibiotic and intensive care for achieving an optimal clinical condition for surgical intervention seem to be unfeasible. In contrast, Sohail et al. identified a subgroup of patients with SAPVE in a previous retrospective and unicentric study, suggesting that exclusive clinical management could potentially prevent in-hospital mortality. This small group (4 patients) was characterized by age < 50 years with good clinical conditions and no cardiac complications or systemic embolization [9]. In our population, no cases with such a favorable outcome without surgical intervention existed.

Although the surgical indications in this study remain aligned with the 2023 ESC guidelines on infective endocarditis [14], the latter introduced some important modifications: the recommendation for urgent surgery (within 3–5 days) in cases of uncontrolled infection, and the need for discussion within the Endocarditis Team regarding early surgery in certain highly virulent microorganisms, including *Staphylococcus aureus*.

The univariate analysis showed 10 predictors of in-hospital mortality in this population. These predictors can be related to intrinsic features of the agent (*S. aureus* infections), clinical complications (peri-annular abscess, severe heart failure - NYHA class III/IV - related to prosthetic valve dysfunction), laboratorial features (high leukocyte count and C-reactive protein levels), patient profile (older age, previous combined valve replacement surgery with myocardial revascularization, left ventricular dysfunction) and aortic prostheses involvement. The lack of valve replacement surgery was associated with in-hospital mortality. Multivariate analysis reinforces the potential protective role of surgical intervention in staphylococcal EO-PVE, with an 80% relative reduction in in-hospital mortality (OR 0.2, *p* < 0.04). Moreover, multivariate analysis also indicates SAPVE as an independent and strong predictor of in-hospital mortality, emphasizing the high clinical aggressiveness of *S. aureus* compared to other staphylococcal species. As shown in Table 5.

**Table 5:** Multivariate analysis of in-hospital mortality in staphylococcal PVE

	OR	95% CI	p-value
SAPVE	10.2	1.89–55.2	0.006
Prosthetic valve replacement	0.2	0.04–0.93	0.04

CI: Confidence interval; OR: Odds ratio; PVE: Prosthetic valve replacement; SAPVE: *Staphylococcus aureus* prosthetic valve endocarditis

Importantly, no statistically significant differences were observed between the Risk-E subgroup and the overall cohort in terms of demographic, clinical, microbiological, or echocardiographic variables. This finding supports the notion that the subgroup in which the Risk-E score could be calculated is representative of the entire population studied, thereby minimizing the risk of selection bias.

The present study has inherent limitations related to its unicentric and observational design. As a sub-analysis, the available clinical and surgical information was restricted to the original database. Consequently, potential biases may arise due to the lack of data regarding previous sternotomies, operative time, and perioperative complications. In addition, the absence of pre- and postoperative neurological status prevented us from evaluating the timing of surgery in patients with neurological complications. The lack of matching and adjustment for confounders, treatment selection biases, and the absence of randomization further compromise the intergroup comparative analysis. Nevertheless, the findings remain relevant given the relative scarcity of staphylococcal EO-PVE in clinical practice.

## Conclusion

In conclusion, staphylococcal EO-PVE mortality is high, especially in SAPVE, where the majority of deaths were concentrated in non-operated patients. In this group, despite surgical indication, the early onset of septic shock and MODS does not allow immediate surgical intervention. Prosthetic valve replacement was significantly associated with in-hospital survival, and only 5.7% of the study population survived without valve replacement surgery.

## Electronic supplementary material

Below is the link to the electronic supplementary material.

Supplementary Material 1

## Data Availability

The datasets generated and/or analysed during the current study are not publicly available due to patient confidentiality, but are available from the corresponding author on reasonable request.

## Abbreviation

EOPVE: Early-onset prosthetic valve endocarditis
CoNSPVE: Coagulase-negative staphylococci prosthetic valve
SAPVE: Staphylococcus aureus prosthetic valve endocarditis
MODS: Multiple Organ Dysfunction Syndrome

## References

[1] Habib G, Thuny F, Avierinos JF. Prosthetic valve endocarditis: current approach and therapeutic options. Prog Cardiovasc Dis. 2008;50(4):274–81 18156006 10.1016/j.pcad.2007.10.007

[2] Habib G, Lancellotti P, Antunes MJ, et al. Esc guidelines for the management of infective endocarditis: the task force for the management of infective endocarditis of the European society of cardiology (esc) endorsed by: European association for Cardio-thoracic surgery (eacts), the European association of nuclear medicine (EANM). Eur Heart J. 2015 2015;36(44):3075–128 10.1093/eurheartj/ehv319 26320109

[3] Siciliano RF, Randi BA, Gualandro DM, et al. Early-onset prosthetic valve endocarditis definition revisited: prospective study and literature review. Int J Infect Dis. 2018;67:3–6 28935245 10.1016/j.ijid.2017.09.004

[4] Abdallah L, Habib G, Remadi JP, Salaun E, Casalta JP, Tribouilloy C. Comparison of prognoses of staphylococcus aureus left-sided prosthetic endocarditis and prosthetic endocarditis caused by other pathogens. Arch Cardiovasc Dis. 2016;109(10):542–49 27342809 10.1016/j.acvd.2016.02.010

[5] Nataloni M, Pergolini M, Rescigno G, Mocchegiani R. Prosthetic valve endocarditis. J Cardiovasc Med (hagerstown). 2010 Dec;11(12):869–83 20154632 10.2459/JCM.0b013e328336ec9a

[6] Pettersson GB, Hussain ST. Current aats guidelines on surgical treatment of infective endocarditis. Ann Cardiothorac Surg. 2019;8(6):630–44 31832353 10.21037/acs.2019.10.05 PMC6892713

[7] Baumgartner H, Falk V, Bax JJ, De Bonis M, Hamm C, Holm PJ, Iung B, Lancellotti P, Lansac E, Rodriguez Muñoz D, Rosenhek R, Sjögren J, Tornos Mas P, Vahanian A, Walther T, Wendler O, Windecker S, Zamorano JL, ESC Scientific Document Group. ESC/EACTS guidelines for the management of valvular heart disease. Eur Heart J. 2017;38(36):2739–91. 2017 Sep 21 28886619 10.1093/eurheartj/ehx391

[8] López J, Revilla A, Vilacosta I, et al. Definition, clinical profile, microbiological spectrum, and prognostic factors of early-onset prosthetic valve endocarditis. Eur Heart J. 2007;28(6):760–65 17255216 10.1093/eurheartj/ehl486

[9] Sohail MR, Martin KR, Wilson WR, Baddour LM, Harmsen WS, Steckelberg JM. Medical versus surgical management of staphylococcus aureus prosthetic valve endocarditis. Am J Med. 2006;119(2):147–54 16443417 10.1016/j.amjmed.2005.09.037

[10] Galar A, Weil AA, Dudzinski DM, Muñoz P, Siedner MJ. Methicillin-resistant staphylococcus aureus prosthetic valve endocarditis: pathophysiology, epidemiology, clinical presentation, diagnosis, and management. Clin Microbiol Rev. 2019 Feb 13;32(2):e00041–18 30760474 10.1128/CMR.00041-18 PMC6431130

[11] Hoerr V, Franz M, Pletz MW, Diab M, Niemann S, Faber C, Doenst T, Schulze PC, Deinhardt-Emmer S, S LB. Aureus endocarditis: clinical aspects and experimental approaches. Int J Med Microbiol. 2018 Aug;308(6):640–52 29526448 10.1016/j.ijmm.2018.02.004

[12] Murray RJ. Staphylococcus aureus infective endocarditis: diagnosis and management guidelines. Intern Med J. 2005 Dec;35(Suppl 2):S25–44 16271059 10.1111/j.1444-0903.2005.00978.x

[13] Tan HL, Chai LY, Yeo TC, Chia BL, Tambyah PA, Poh KK. Predictors of In-hospital adverse events in patients with prosthetic valve infective endocarditis. Heart Lung Circ. 2015;24(7):705–09 25743477 10.1016/j.hlc.2015.01.013

[14] Delgado V, et al. 2023 esc guidelines for the management of endocarditis: developed by the task force on the management of endocarditis of the European society of cardiology (esc) endorsed by the European association for Cardio-thoracic surgery (eacts) and the European association of nuclear medicine (EANM). Eur Heart J. 2023;44(39):3948–4042. 10.1093/eurheartj/ehad193 10.1093/eurheartj/ehad193 37622656

